# Role of m6A methyltransferase component VIRMA in multiple human cancers (Review)

**DOI:** 10.1186/s12935-021-01868-1

**Published:** 2021-03-17

**Authors:** Wei Zhu, Jing-Zi Wang, Ji-Fu Wei, Chen Lu

**Affiliations:** 1 Precision Medicine Center, First Affiliated Hospital of Gannan Medical University, 128 Jinling Road, Ganzhou, 341000 China; 2 Department of Urology, Children’s Hospital of Nanjing Medical University, 72 Guangzhou Road, Nanjing, 210029 Jiangsu China; 3 Research Division of Clinical Pharmacology, The First Affiliated Hospital of Nanjing Medical University (Jiangsu Province Hospital), 300 Guangzhou Road, Nanjing, 210029 Jiangsu China

**Keywords:** VIRMA, KIAA1429, M6A, Cancer, Therapy

## Abstract

*N*6-Methyladenosine (m6A) modification is one of the most widely distributed RNA modifications in eukaryotes. It participates in various RNA functions and plays vital roles in tissue development, stem cell formation and differentiation, heat shock response control, and circadian clock controlling, particularly during tumor development. The reversible regulation of m6A modification is affected by the so-called ‘reader’, ‘writer’ and ‘eraser’. As a required component and the largest methyltransferase, vir-like m6A methyltransferase associated (VIRMA) can promote the progression of cancer and is associated with poor survival in multiple types of cancer. The present review investigated the role of VIRMA in various types of cancer. In an m6A-dependent or -independent manner, VIRMA can play an oncogenic role by regulating cancer cell proliferation, migration and invasion, metastasis, apoptosis resistance and tumor growth in different pathways by targeting stem factors, CCAT1/2, ID2, GATA3, CDK1, c-Jun, etc. VIRMA can also predict better prognosis in kidney renal clear cell carcinoma (KIRC), kidney renal papillary cell carcinoma (KIRP) and papillary thyroid carcinoma by TCGA analysis. The obvious oncogenic roles of VIRMA observed in different types of cancer and the mechanisms of VIRMA promoting cancers provided the basis for potential therapeutic targeting for cancer treatments.

## Background

Chemical modification of nucleic acids is an important part of biological processes, including RNA transcription, protein translation and signaling pathway. *N*6-Methyladenosine (m6A) modification is one of the most widely distributed RNA modifications in eukaryotes [1, 2]. m6A modification refers to methylation occurring at the sixth position of nitrogen atoms of adenosine at the post-transcriptional level, with *S*-adenosylmethionine serving as the methyl donor for m6A formation [3–5]. m6A modification exists in mammalian mRNAs [6, 7], long-noncoding RNAs [8], and microRNAs [9, 10], and participates in various RNA functions such as mRNA stability [6, 11], splicing [12], transport [13], translation [7, 14], primary microRNA processing [9] and RNA–protein interactions [15].

**Fig. 1:**
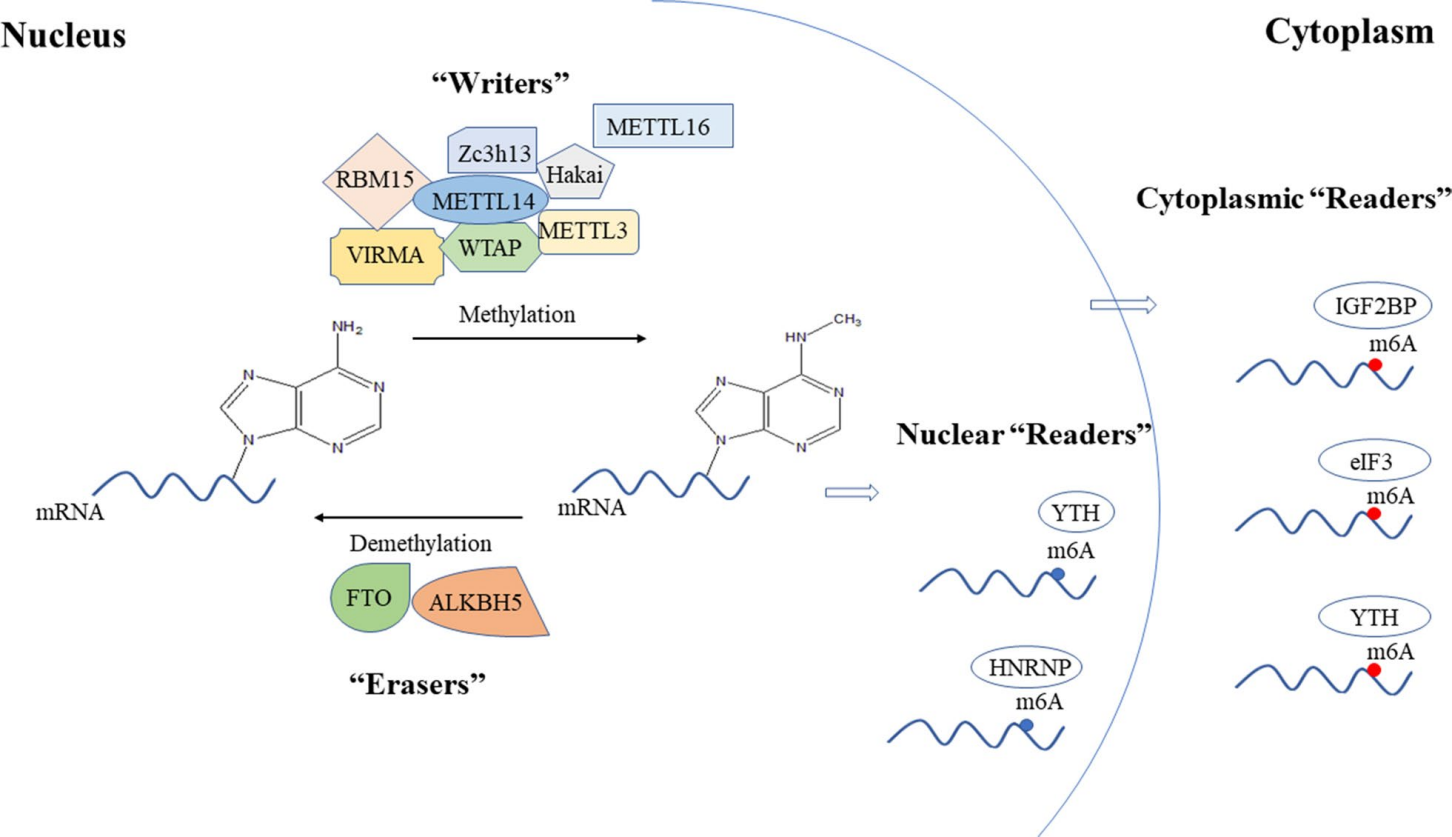
Roles of the *N*6-methyladenosine ‘writer’, ‘eraser’ and ‘reader’ complexes in regulating mRNA. *METTL3* methyltransferase like 3, *METTL14* methyltransferase like 14, *WTAP* Wilms tumor 1-associated protein, *VIRMA* vir-like m6A methyltransferase associated, *METTL16* methyltransferase like 16, *ZC3H13* zinc finger CCCH-type containing 13, *RBM15* RNA binding motif protein 15, *ALKBH5* alkB homolog 5, *FTO* the fat mass and obesity-associated protein, *IGF2BP* IGF2 mRNA binding proteins, *eIF3* eukaryotic translation initiation factor 3, *HNRNP* heterogeneous nuclear ribonucleoprotein, *YTH* YT521-B homology domain-containing protein

The reversible regulation of m6A modification is performed by the so-called ‘reader’, ‘writer’ and ‘eraser’ [16, 17] (Fig. 1). The ‘writers’ are also called methyltransferases, including methyltransferase like 3 (METTL3) [18], METTL14 [18], Wilms’ tumor 1-associating protein (WTAP) [19], vir-like m6A methyltransferase associated (VIRMA; also known as KIAA1429) [18, 20], METTL16 [21], RNA-binding motif protein 15 (RBM15) [22, 23], zinc finger CCCH-type containing protein 13 (Zc3h13) [22] and Hakai [24]. Studies have shown that m6A modification plays a vital role in tissue development, stem cell formation and differentiation [25, 26], heat shock response control [27] and circadian clock control [28], particularly during tumor development.

The largest component of ‘writers’, VIRMA, has been verified to promote cancer progression or is associated with poor survival in multiple cancer types, including liver cancer [29], gastric cancer [30] and breast cancer [20], head and neck squamous cell carcinoma (HNSC) [31], and testicular germ cell tumors (TGCT) [32] and etc. VIRMA can participate in cancer progression in an m6A-dependent manner or m6A-independent manner. The obvious oncogenic roles of VIRMA observed in these cancer types suggested that VIRMA may serve as a potential therapeutic target in cancer treatment.

## Main text

### VIRMA

**Fig. 2:**
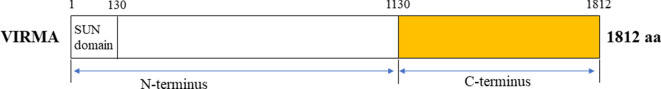
Schematic of domain architecture of VIRMA. *aa* amino acids; N-terminus (1–1130 aa, N-VIRMA); C-terminus (1131–1812 aa, C-VIRMA)

It was first reported that VIRMA is required for the entire process of mRNA methylation in human cells in 2015 [18]. As a VIRMA homologue in *Drosophila melanogaster*, virilizer plays an essential role in sex-lethal splicing, male and female viability, and the capability of eggs production in embryonic development [33]. In human, VIRMA is located in the nuclear speckles, the same location as that of WTAP [34, 35]. As the largest known component of the methyltransferase complex (202 kDa), VIRMA contains an N-terminal (1130 aa) as N-VIRMA and a C-terminal (1131–1812 aa) as C-VIRMA, and begins from a SUN domain (130 aa) (Fig. 2) [22, 36, 37]. A fourfold decrease in m6A peak scores has been reported following VIRMA knockdown, more obvious than that observed in human cells following METTL3 and METTL14 knockdown [18]. It was reported that in human HeLa cells, VIRMA could recruit the m6A methyltransferase components METTL3/METTL14/WTAP to guide region-selective methylations [38], suggesting that VIRMA plays a significant role in m6A modification. The m6A modification by VIRMA is enriched in the 3′-untranslated region (3′-UTR) and near the stop codon of RNA substrates [20, 38]. Virilizer contains an RNA-binding protein (RBP) domain, similar with that in RNA and DNA helicases, and ribonucleoproteins, and is involved in the RNA processes of metabolism, transport and translation [33]. Studies have demonstrated that the abnormal expression of RBPs could lead to the upregulation of certain oncogenes or the downregulation of certain tumor suppressor genes [39]. VIRMA has been reported to mainly act as the oncogene in several types of cancer, as listed in Table 1. Wang et al. [40] found that hsa_circ_0084922 (also named circ_KIAA1429), coming from VIRMA, was upregulated in hepatocellular carcinoma (HCC), which may promote cancer progression.

**Table 1:** Expression, clinical significance, and biological functions of VIRMA in various cancers

Cancer type	Expression	Clinical significance	Biological functions	Methodology	In vivo or in vitro	References
Breast cancer	Upregulated (46 pairs)	Oncogene	Proliferation, metastasis, tumorigenesis	mRNA high-throughput sequencing; RIP-seq; m6A dot blot assay; mRNA FISH; mRNA stability analysis;	In vivo and in vitro	[20]
	Upregulated (1109 BRC tissues and 113 normal breast tissues from TCGA; TMA containing 20 BRC specimens and 20 normal breast tissue specimens)	–	–	TMA cohorts; Immunohistochemical staining; Immunofluorescence assay	–	[42]
	Upregulated (~ 7400 samples representing 11 cancer types)	–	–	–	–	[41]
Liver cancer	Upregulated (70 pairs)	Oncogene	Cell proliferation, tumor growth and metastasis, cell apoptosis resistance	RNA-seq; RIP-seq; MeRIP-seq	In vivo and in vitro	[29]
	Upregulated (371 liver cancer tissues and 50 normal liver tissues from TCGA)	Oncogene	Cell proliferation, cell migration and invasion	MeRIP-PCR	In vitro	[43]
	Upregulated (374 HCC and 50 normal tissues from TCGA; 243 HCC and 202 normal tissues from ICGC)	Oncogene	–	–	–	[45]
Gastric cancer	Upregulated (20 pairs)	Oncogene	Proliferation	Dual‐luciferase reporter gene assay; RIP; RNA-seq	In vivo and in vitro	[30]
	Upregulated (~ 7400 samples representing 11 cancer types)	–	–	–	–	[41]
Kidney cancer	Upregulated	Oncogene	–	–	–	[44]
		–	–	–	–	[41]
Kidney renal papillary cell carcinoma	Downregulated (289 kidney renal papillary cell carcinoma and 32 normal kidney samples from the TCGA database)	–	–	–	–	[49]
Bladder cancer	–	Oncogene	–	–	–	[44]
	No difference (408 bladder cancer patients from TCGA)	Oncogene	–	–	–	[48]
Prostate cancer	Upregulated (TCGA + GTEx, 492 prostate adenocarcinoma and 152 adjacent normal specimens)	Oncogene	Proliferation, metastasis	RNA methylation quantification; immunofluorescence Analysis; RT2 lncRNA PCR Array Human Cancer PathwayFinder; *N*6-methyladenosine immunoprecipitation	In vitro	[58]
	Upregulated	–	–	–	–	[44]
Testicular cancer	Upregulated	Oncogene	–	qRT-PCR; Immunohistochemistry	In vitro	[32]
	Upregulated	Oncogene	–	–	–	[44]
Head and neck squamous cell carcinoma	TCGA	–	–	–	–	[31]
Osteosarcoma	Upregulated	Oncogene	Proliferation, invasion, and migration	Tissue microarray (TMA) construction and immunohistochemistry (IHC); dual luciferase reporter gene assay	In vivo and in vitro	[50]
Lung squamous cell carcinoma	Upregulated (~ 7400 samples representing 11 cancer types)	–	–	–	–	[41]
Colon adenocarcinoma	Upregulated (~ 7400 samples representing 11 cancer types)	–	–	–	–	[41]
Rectum adenocarcinoma	Upregulated (~ 7400 samples representing 11 cancer types)	–	–	–	–	[41]
Lung adenocarcinoma	Upregulated (~ 7400 samples representing 11 cancer types)	–	–	–	–	[41]
Cholangiocarcinoma	Upregulated (~ 7400 samples representing 11 cancer types)	–	–	–	–	[41]
Uterine corpus endometrial carcinoma	Downregulated (~ 7400 samples representing 11 cancer types)	–	–	–	–	[41]
Thyroid carcinoma	Downregulated (~ 7400 samples representing 11 cancer types)	–	–	–	–	[41]
papillary thyroid carcinoma	Downregulated (499 tumor tissues and 58 normal thyroid carcinoma)	Tumor suppressor gene	–	–	–	[52]
Ovarian cancer	Downregulated (TCGA, 370 ovarian cancer tissues and 88 normal tissues)	–	–	–	–	[51]

TCGA: http://cancergenome.nih.gov/

ICGC: https://dcc.icgc.org/releases/current/Projects/LIRI-JP

GTEx: https://gtexportal.org/

### VIRMA in cancers

m6A regulators, including ‘writers’, ‘erasers’, ‘readers’, play key roles in cancer. VIRMA was found to be correlated with the most positive oncogenic pathways among the ‘writers’ [41], indicating that VIRMA may has multiple different functions and plays important roles in cancer pathways.

#### Expression of VIRMA in cancers

The difference of VIRMA expression among different tissues was shown in Li’s study [41]. The analysis revealed that VIRMA expression was higher in HNSC, lung squamous cell carcinoma (LUSC), liver hepatocellular carcinoma (LIHC), colon adenocarcinoma (COAD), rectum adenocarcinoma (READ), lung adenocarcinoma (LUAD), stomach adenocarcinoma (STAD), cholangiocarcinoma (CHOL), breast invasive carcinoma (BRCA), kidney chromophobe (KICH) and kidney renal clear cell carcinoma (KIRC). VIRMA was more lowly expressed in uterine corpus endometrial carcinoma (UCEC), thyroid carcinoma (THCA), prostate adenocarcinoma (PRAD) and kidney renal papillary cell carcinoma (KIRP).

#### Prognostic potential of VIRMA in cancer

To investigate the association between VIRMA expression and cancer prognosis, the impact of VIRMA expression on survival rate was determined by Kaplan–Meier survival curves. VIRMA was found to be highly expressed and to predict poor overall survival (OS) in breast cancer [20, 41, 42], liver cancer [29, 43] and kidney cancer [44] (Table 2). However, the high VIRMA expression predicted the better OS in KIRC [41] (Table 2). In addition, VIRMA was highly expressed and predicted poor disease-free survival (DFS) in liver cancer [29] and kidney cancer (Table 2) [44].

**Table 2:** Survival influence of VIRMA in various cancers

Cancer type	Sample size	Survival influence	References
Breast cancer	low-VIRMA (n = 528); high-VIRMA (n = 528)	Worse OS	[20]
	low-VIRMA (n = 598); high-VIRMA (n = 472)	Worse OS	[42]
	low-VIRMA (n = 611); high-VIRMA (n = 457)	Worse RFS	[42]
	TCGA	Worse OS	[41]
Liver cancer	70 HCC patients	Worse OS and DFS	[29]
	low-VIRMA (n = 182); high-VIRMA (n = 182)	Worse OS	[43]
	374 HCC and 50 normal tissues from TCGA; 243 HCC and 202 normal tissues from ICGC	Worse OS	[45]
Kidney cancer	897 samples from 895 patients	Worse OS and DFS	[44]
Kidney renal clear cell carcinoma	TCGA	Better OS	[41]
Kidney renal papillary cell carcinoma	289 kidney renal papillary cell carcinoma and 32 normal kidney samples from the TCGA database	Better OS and DFS	[49]
Prostate cancer	TCGA + GTEx, 492 prostate adenocarcinoma and 152 adjacent normal specimens	Worse DFS	[58]
Gliomas	309 glioma patients from CGGA and 595 glioma patients from TCGA	Worse OS	[75]
Ovarian cancer	370 ovarian cancer tissues and 88 normal tissues from TCGA	Worse OS	[51]
Papillary thyroid carcinoma	499 tumor tissues and 58 normal thyroid carcinoma	Better OS	[52]

TCGA: http://cancergenome.nih.gov/

CGGA: www.cgga.org.cn

ICGC: https://dcc.icgc.org/releases/current/Projects/LIRI-JP

GTEx: https://gtexportal.org/

#### Alteration frequency of VIRMA in 33 cancer types

**Fig. 3:**
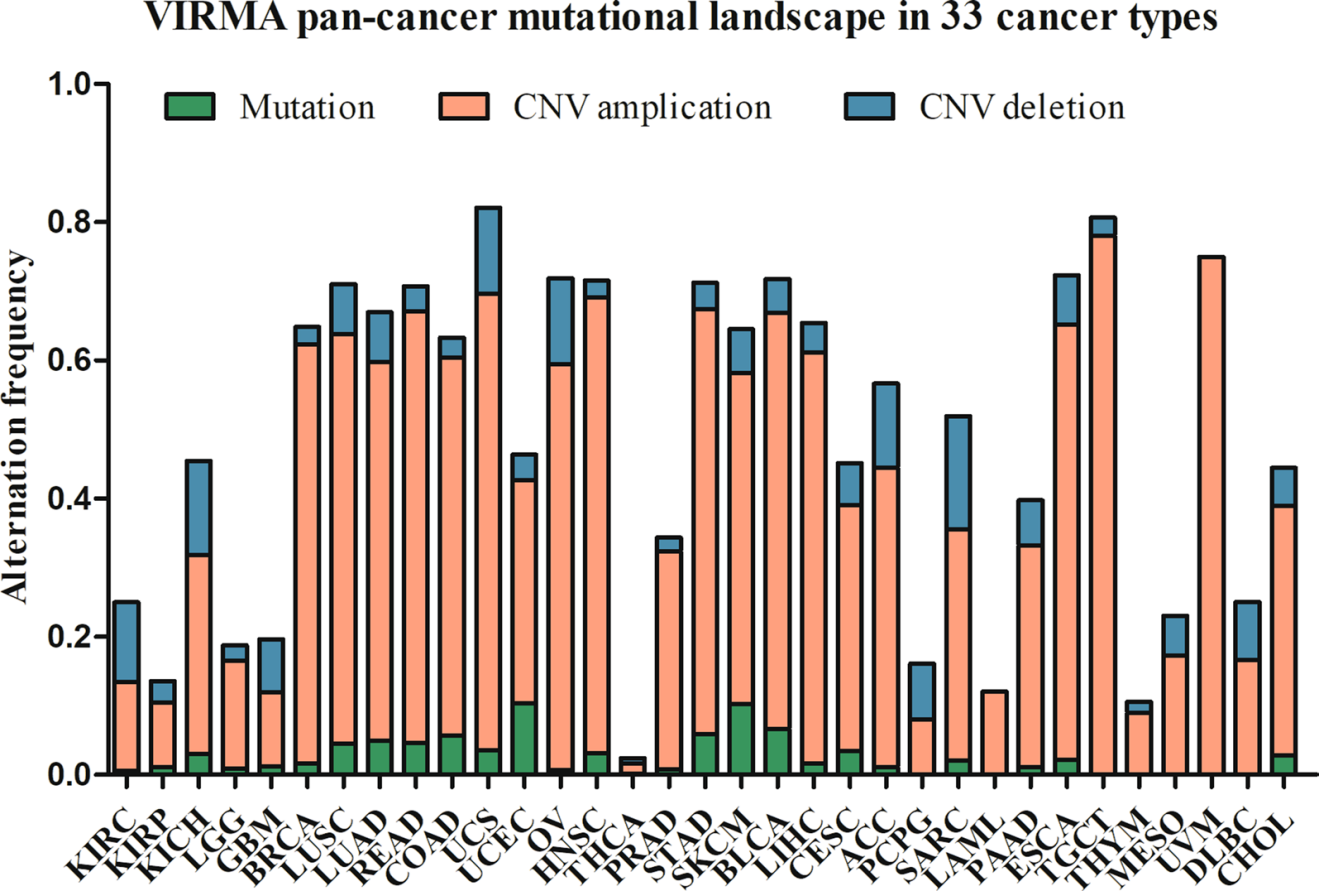
VIRMA pan-cancer mutational landscape in 33 cancer types. *CNV* copy number variation, *KIRC* kidney renal clear cell carcinoma, *KIRP* kidney renal papillary cell carcinoma, *KICH* kidney chromophobe, *LGG* brain lower grade Glioma, *GBM* glioblastoma multiforme, *BRCA* breast invasive carcinoma, *LUSC* lung squamous cell carcinoma, *LUAD* lung adenocarcinoma, *READ* rectum adenocarcinoma, *COAD* colon adenocarcinoma, *UCS* uterine carcinosarcoma, *UCEC* uterine corpus endometrial carcinoma, *OV* ovarian serous cystadenocarcinoma, *HNSC* head and neck squamous carcinoma, *THCA* thyroid carcinoma, *PRAD* prostate adenocarcinoma, *STAD* stomach adenocarcinoma, *SKCM* skin cutaneous melanoma, *BLCA* bladder urothelial carcinoma, *LIHC* liver hepatocellular carcinoma, *CESC* cervical squamous cell carcinoma and endocervical adenocarcinoma, *ACC* adrenocortical carcinoma, *PCPG* pheochromocytoma and paraganglioma, *SARC* sarcoma, *LAML* acute myeloid leukemia, *PAAD* pancreatic adenocarcinoma, *ESCA* esophageal carcinoma, *TGCT* testicular germ cell tumors, *THYM* thymoma, *MESO* mesothelioma, *UVM* uveal melanoma, *DLBC* lymphoid neoplasm diffuse large B-cell lymphoma, *CHOL* cholangiocarcinoma

To explore the alteration frequency of VIRMA in 33 cancers, we extracted the result data from the study by Li et al. [42]. The original data in Li’s study was from TCGA MAF file (“MC3”) and Broad GDAC Firehose (https://gdac.broadinstitute.org/) [41]. The overall copy number variation (CNV) amplification frequency of VIRMA in cancer was 1.40–78%, with a high frequency in most tumors, such as uveal melanoma (UVM), TGCT, and low frequency in THCA, PCPG (Fig. 3) [41]. The overall CNV deletion frequency of VIRMA in cancers was 0–16.34%, with high frequency in sarcoma (SARC), kidney Chromophobe (KICH), and low frequency in UVM, THCA (Fig. 3) [41]. The overall VIRMA mutation frequency in cancers was 0–10.35%, with a high frequency in SKCM and UCEC, and low frequency in lymphoid neoplasm diffuse large B-cell lymphoma (DLBC), UVM, mesothelioma (MESO), THTM and TGCT (Fig. 3).

#### VIRMA in breast cancer

The functions of VIRMA in breast cancer tumorigenesis and the related mechanisms have been previously reported [20]. VIRMA was found to be more highly expressed in breast cancer tissues than in non-tumor breast tissues in clinical samples [20, 42]. The high expression of VIRMA also predicted poor OS in patients with breast cancer [20, 42]. VIRMA could promote breast cancer proliferation and metastasis in vivo and in vitro, which indicated that VIRMA could promote breast cancer progression and was associated with pathogenesis. In addition, 5′-fluorouracil (5-FU) could decrease the expression of VIRMA and its downstream target in breast cancer cells [20].

#### IRMA and circ_KIAA1429 in liver cancer

A higher expression of VIRMA was observed in liver cancer tissues, as compared with adjacent normal tissues, both in the TCGA database and clinical samples, which predicted poor OS and DFS in patients with liver cancer [29, 43, 45]. Knockdown of VIRMA could inhibit cancer cell proliferation and metastasis in vitro [43]. Lan et al. [29] further demonstrated that VIRMA markedly facilitated the cell cycle progression, cell proliferation, invasion, and migration, as well as apoptosis resistance in vitro. It also promoted tumor growth, as well as pulmonary and intrahepatic metastasis in vivo, which confirmed and identified VIRMA as a formidable driver of liver tumor growth and metastasis. To further understand whether the differential expression of VIRMA is caused by genetic changes related to the corresponding genes, Qu et al. [45] then analyzed CNV and single-nucleotide polymorphism (SNP) data from the TCGA database and found that the CNV in HCC tissues was significantly different from that in normal tissues. More specifically, VIRMA mainly exhibited increased copy numbers in HCC tissues [45]. It was also observed that SNP mutations was very low in HCC tissues, which indicated that upregulation of VIRMA is not entirely caused by CNV or SNP mutations in the corresponding genes [45].

circ_KIAA1429 (chr8:95547066–95550574, 305 bp spliced sequence, http://www.circbase.org/), which comes from VIRMA, was obviously upregulated in HCC cancer cells and tissues. Wang et al. [40] demonstrated that the upregulation of circ_KIAA1429 could promote migration, invasion and EMT in HCC cancer cells in vitro and in vivo, whereas the knockdown of circ_KIAA1429 yielded the opposite results.

#### VIRMA in gastric cancer

A higher expression of VIRMA was observed in gastric cancer tissues, as compared with their adjusted normal tissues, which was associated with tumor grade. The knockdown of VIRMA could inhibit gastric cancer cell proliferation by arresting the cell cycle in vivo and in vitro. Miao et al. [30] demonstrated that VIRMA promotes gastric cancer, which may serve as a therapeutic target in the future.

#### VIRMA in urological cancers

VIRMA was upregulated in four major urogenital neoplasms: including kidney cancer, bladder cancer, prostate cancer and testicular cancer [44]. TGCT account for > 95% of all types of testicular neoplasms, and consist of two major families: the germ-cell neoplasia in situ (GCNIS)-related tumors [the most frequent; include seminomas (SEs) and non-seminomatous tumors (NSTs), which have an obvious different clinical behavior and impact], and the GCNIS-unrelated ones [46].

In prostate cancer, the high expression of VIRMA was correlated with cancer progression. In TGCTs, VIRMA appeared to be a promising biomarker for distinguishing between SEs and NSTs, and had an impact disease stage [32, 44]. In bladder cancer, VIRMA seems to be a useful marker, as it is amongst the most commonly deregulated and significantly upregulated in high grade tumors [44]. VIRMA was significantly upregulated in non-papillary tumors (the most aggressive, more prone to progress and metastasize) among different types of bladder cancers [47]. Chen. et al. [48] latter confirmed that VIRMA was highly expressed in high grade bladder cancer and further found that the expression of VIRMA did not differ between BC and normal tissues. According to the current World Health Organization (WHO) 2016 classification, kidney cancer (also named renal cell carcinoma, RCC) are mainly classified into three subtypes: KICH, KIRC and KIRP [46]. In kidney cancer, the VIRMA expression could be a biomarker to discriminate these RCC subtypes, and was associated with OS and DFS [44]. Sun et al. further focused on KIRP study and found that VIRMA was downregulated in, compared with in normal kidney tissue samples [49]. High expression of VIRMA was correlated with high grade of KIRP and predicted poor OS and DFS, which indicated that VIRMA is a potential prognostic biomarker that can accurately predict survival outcomes of KIRP patients [49]. However, the data of the above studies were from TCGA database, that needs the validation of clinical results and further mechanism research, especially to explain the different results between kidney cancer and KIRP need further clinical confirm and further mechanism research.

Of note, VIRMA and m6A methylation reader YTHDF3 were upregulated and showed a strong positive correlation in prostate cancer and TGCTs, as they jointly facilitated poor prognosis [32, 44].

#### HNSC

VIRMA was more highly expressed in HNSC tissues, as compared with their adjacent normal tissues, as shown by TCGA analysis [31]. The result was from TCGA database, which need clinical confirm and mechanism research.

#### VIRMA in osteosarcoma

VIRMA was upregulated and associated with unfavorable outcomes in patients with osteosarcoma. Han et al. [50] revealed that knockdown of VIRMA could suppress osteosarcoma cancer cell migration, invasion, and proliferation in vitro, as well as suppress tumor growth in vivo. It was also found that VIRMA was the direct target of miR-143, and could partly reverse the anti-proliferative effect of miR-143 [50]. miR-143 and VIRMA may become potential predictive biomarkers in the treatment of osteosarcoma.

#### VIRMA in ovarian cancer

Ovarian cancer is one of the deadliest gynecological malignancies. VIRMA protein was found to be downregulated in ovarian cancer tissues than normal tissues and positively correlated with worse OS [51]. The expression of VIRMA in different types of ovarian cancers in the Oncomine database is different and the highest in ovarian serous adenocarcinoma [51]. The expression of VIRMA in grade 3 is significantly higher than that in grade 2 [51]. The samples in grade 1 and grade 4 were insufficient, which need further collected and analyzed. However, the protein expression and the mRNA expression of VIRMA in the normal control group and tumor group was not consistent, which suggests that the mRNA may serve as a reservation [51]. Only under certain circumstances, such as hypoxia or immune stimulation, can the protein be translated. A similar phenomenon could also be observed in the production of some cytokines [51]. The mRNA may also regulate the expression of other proteins by generating microRNAs. The studies on ovarian cancer were from TCGA database, which need further experiments and analysis in detail.

#### VIRMA in papillary thyroid carcinoma

VIRMA was downregulated and predicted better OS in papillary thyroid carcinoma [41, 52]. Univariate and multivariate analyses demonstrated that the risk score of VIRMA is an independent prognostic factor in papillary thyroid carcinoma, indicating that VIRMA might act as a tumor suppressor [52]. Studies on VIRMA in papillary thyroid carcinoma were from TCGA database, which need further exploration of clinical confirm and the underlying molecular mechanisms.

### Regulatory mechanisms of VIRMA in cancer

**Fig. 4:**
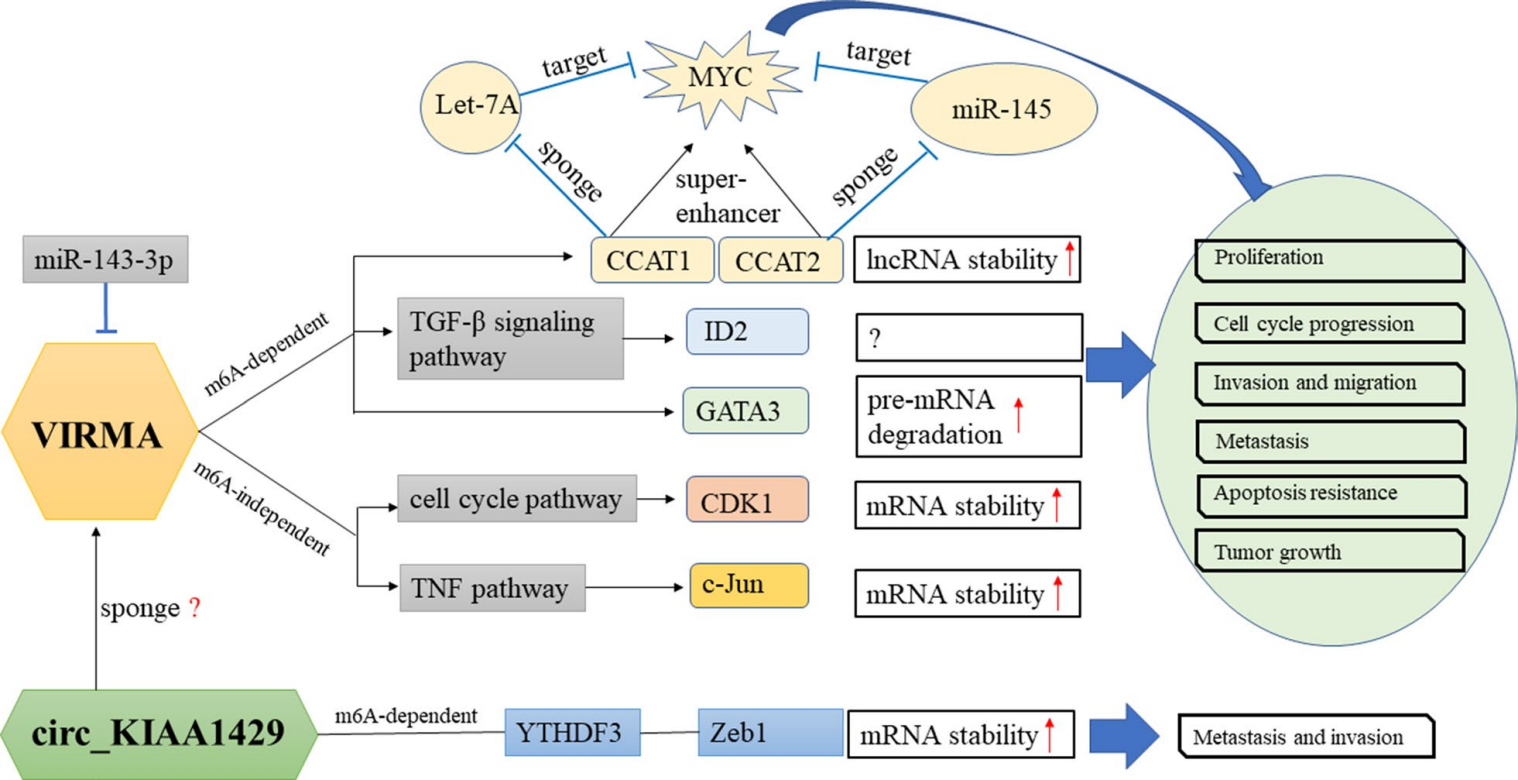
Mechanisms of VIRMA and circ_KIAA1429 involved in human cancer progression. VIRMA expression could be regulated by miR-143-3p. VIRMA played an important role in tumor cell proliferation, invasion, migration, cell cycle, metastasis, apoptosis resistance, tumorigenesis in an m6A-dependent or m6A-independent manner. The TGF-β, cell cycle, TNF and other signaling pathways were involved in the underlying mechanism. Circ_KIAA1429 can promote HCC through the mechanism of m6A-YTHDF3-Zeb1 pathway. VIRMA, vir-like m6A methyltransferase associated

As shown in Fig. 4, several m6A modification regulators participate in cancer development in an m6A-dependent manner by targeting related mRNAs. As regards the regulatory role of VIRMA in cancer, it was found that, in addition to the m6A-dependent pathway, VIRMA could also regulate downstream m6A-independent pathways. Circ_KIAA1429 could also regulate downstream m6A-dependent pathways. The expression of VIRMA may also be regulated by miR-143-3p in cancer cells.

#### VIRMA regulates downstream targets in an m6A-dependent manner

In the VIRMA-depleted cell lines, m6A modification around the 3′-UTR and near the stop codon significantly disappeared, indicating that VIRMA can exert its effects by mediating mRNA m6A methylation in 3′UTR and near stop codon region [38]. Yue et al. [38] revealed VIRMA functioned by recruiting the methyltransferase core components and interacting with polyadenylation cleavage factors CPSF5 and CPSF6, which suggested interactions between the m6A methylation and polyadenylation during mRNA processing and mRNA metabolism.

##### ID2 target

VIRMA promoted cell migration and invasion in liver cancer by increasing the m6A modification of ID2 mRNA, which then led to the decrease in ID2 expression [43]. Decreasing ID2 could regulate the secretion of vascular endothelial growth factor to promote liver cancer metastasis [53]. The ID gene family, including ID2, was found to be upregulated in various kinds of cancers [54, 55], and ID2 in particular was associated with the development of several diseases. VIRMA regulated the migration and invasion of liver cancer by regulating the m6A modification of ID2.

##### GATA3 target

GATA3 was identified as the direct downstream target of VIRMA-mediated m6A modification in liver cancer through the combination of immunoprecipitation sequencing (RIP-seq), and high-throughput methylated RNA immunoprecipitation sequencing (MeRIP-seq) [29]. VIRMA methylate in the 3′-UTR of GATA3 pre-mRNA to separate the RNA-binding protein HuR and promote GATA3 pre-mRNA degradation. GATA3-AS, a long-noncoding RNA (lncRNA), transcribed from the antisense strand of the GATA3 gene, acts as a cis-acting element in the interaction between VIRMA and GATA3 pre-mRNA. Therefore, VIRMA promote the growth, metastasis and malignant phenotypes of liver cancer cells by regulating the GATA expression, which is mediated by the m6A modification of GATA3 pre-mRNA [29].

#### lncRNA target

lncRNAs are important factors in prostate cancer. m6A is considered one of the most prevalent modifications in lncRNAs [56, 57]. VIRMA knockdown could decrease the m6A levels and stability of CCAT1 and CCAT2 lncRNA in prostate cancer. Another study demonstrated that knockdown of VIRMA could decrease the stability of CCAT1 and CCAT2 lncRNA in an m6A-dependent manner to regulate MYC transcription, promoting progression of prostate cancer [58]. A direct correlation between CCAT1/2 and MYC transcript levels was found in prostate cancer [59]. Therefore, Daniela et al. hypothesized that stabilization of lncRNAs CCAT1/2 by m6A modification amplified the effect of MYC expression levels in cancer cells by 2 separate mechanisms: (i) directly, both lncRNAs acted as super-enhancers for positive regulation of MYC mRNA [60]; (ii) indirectly, CCAT1/2 actedas microRNA sponges for MYC-targeting microRNAs let-7A and miR-145, respectively [61–64].

##### Circ_KIAA1429 and Zeb1

CircRNAs have attracted considerable attention in multiple cancer studies in recent years. Overexpression of circ_KIAA1429 could promote HCC cancer cell migration and invasion, whereas the knockdown of circ_KIAA1429 could inhibit these effects. Zeb1 was identified as the downstream target of circ_KIAA1429. It was also found that m6A reader YTHDF3 could stabilize Zeb1 mRNA and prolong its half-life. Overall, circ_KIAA1429 could promote HCC progression through the m6A-YTHDF3-Zeb1 pathway to stabilize Zeb1 expression, which may represent a new target in cancer treatment [40]. As mentioned above, VIRMA is also upregulated and promote cancer progression in HCC [29, 43]. The present study has not clarified whether circ_KIAA1429 affects the expression, biological function, and tumorigenesis effect of VIRMA. Previous studies showed circRNAs might act as a sponge to increase the expression of its host gene [65–67], which would provide possible pathways that how circ_KIAA1429 influence VIRMA expression.

#### VIRMA regulates downstream target in an m6A-independent manner

##### CDK1 target

The cell cycle pathway played a significant role in the oncogenic activities of VIRMA, and ranked in the top two in breast cancer cell RIP-seq [20]. Among cell cycle-related proteins, CDK1 acts as an oncogene in cancers and is most associated with VIRMA in different breast cells. Reverse experiments, RIP-seq, and RT-qPCR confirmed that CDK1 is the main target of VIRMA in breast cancer. METTL3 knockdown could decrease CDK1 mRNAs with m6A modification, while VIRMA knockdown did not change the level of m6A modification in CDK1 mRNAs, indicating that m6A modification did not disturb the interaction between VIRMA and CDK1 in cancer cells [20]. VIRMA facilitated breast cancer by regulating the CDK1 mRNA expression in an m6A-independent manner [20].

##### c-Jun target

It was revealed that transcripts in gastric cancer cells were most enriched in the TNF signaling pathway, as compared with several other cancer‐related pathways, as shown by the KEGG pathway analysis in MGC803 cells [30]. After combining the associated genes by RIP-seq and mRNA-seq, c-Jun was identified as potential downstream target directly regulated by VIRMA in gastric cancer [20, 38]. c‐Jun, a transcriptional activator and member of the AP‐1 family, has been shown to participate in cell proliferation and apoptosis, tumorigenesis, and tissue morphogenesis [68–70]. As the regulation of CDK1 mRNA in breast cancer, no significant difference was observed in luciferase activity between the reporter carrying mutant m6A site of 3′-UTR in c-Jun and that carrying the non-mutant m6A site. It was demonstrated that VIRMA promoted gastric cell progression mainly by directly binding to the 3′-UTR of c‐Jun mRNA to regulate c‐Jun expression in an RNA-binding activity rather than the m6A-dependent manner [30]. However, more experiments should be completed to verify the m6A-independent manner, such as whether METTL3 affect the m6A modification level of c-Jun mRNA in gastric cancer cells.

#### Others

The Notch signaling pathway plays an important role in cell development and differentiation. In recent years, accumulating evidence have suggested that abnormal activation of the Notch signaling pathway participate in tumor progression. The high expression of the Notch signaling pathway has been observed in several types of cancer, an expression that results in Notch signal enhancement, which promotes cancer cell survival [71]. In osteosarcoma, upregulation of VIRMA was found to be associated with the activation of the Notch signaling pathway. It was revealed that VIRMA knockdown could suppress the expression of stem factors Notch1, Oct4, Nanog and CD44 in osteosarcoma cells, which suggested that VIRMA promoted osteosarcoma progression by activating Notch signaling pathway [50]. However, that study did not elucidate whether VIRMA regulates these downstream targets in an m6A-dependent manner.

A study by Han et al. revealed that VIRMA and miR-143-3p were negatively correlated in osteosarcoma samples [50]. The overexpression of miR-143-3p could inhibit VIRMA expression in osteosarcoma cells. The overexpression of VIRMA could reverse the anti-proliferation caused by miR-143-3p. It was proven that miR-143-3p could suppress VIRMA expression by directly targeting its 3ʹ-UTR region in osteosarcoma cells [50].

### Prospect

While the roles of m6A modifications in cancers have been extensively reviewed elsewhere, the crucial functions of VIRMA in various types of cancer, as well as the potential targeting of VIRMA as cancer treatment, have not yet been highlighted. Our review is to summarize and analyze the present research on VIRMA, provide more ideas for the future research.

More studies are needed to demonstrate the biological function of VIRMA and its impact on tumor progression and survival, as well as the mechanisms involved, in more cancer types and phenotypes. High expression of VIRMA has been verified to predict poor survival in multiple cancers, such as breast cancer [20, 41, 42], liver cancer [29, 43], kidney cancer [44] and prostate cancer [58]. The expression of VIRMA is up-regulated in KIRC, but it indicates a poor prognosis. The study did not explain the reason for this conflict, which need further exploration [41]. VIRMA not only regulates the expression of downstream mRNA, but also regulates the stability of pre-mRNAs and lncRNAs. Inhibiting these downstream targets could reverse the oncogenic effect of VIRMA in cancer progression. Generally, VIRMA is an oncogene in most types of cancer, and downregulating it could inhibit cancer progression, indicating that VIRMA can become a diagnostic/prognostic biomarker in certain cancer types. Based on the emerging evidence of the roles and the molecular mechanisms in cancers, m6A regulators have attracted growing investigation as therapeutic targets [72]. Given the critical roles of the m6A regulatory proteins in cancers, m6A modification ‘eraser’ FTO appears to be a good drug target in cancer therapy. Several FTO small-molecule inhibitors, including meclofenamic acid [73] and MO-I-500 [74] have shown effective activity to inhibit the survival and growth of GBM and breast cancer cells by inhibiting the catalytic activity of FTO. Thus, inhibiting VIRMA and these downstream targets are considered potent cancer therapies. Studies have not yet elucidated the mechanism underlying the downstream regulation of VIRMA and whether it occurs through the m6A pathway in several tumor models, which is crucial for the development of treatments. The underlying mechanisms of VIRMA in cancer should be further addressed. Novel therapeutic strategies for m6A RNA methylation should be further explored in the treatment of cancer.

## Conclusions

m6A modification has been shown to act by influencing RNA transcript, splicing, processing, translation and decay, and to participate in the development of various cancer types [6, 7, 9, 11–15]. VIRMA is a required component of the methyltransferase complex in m6A modification. It mediates methylation in the 3′-UTR and near the stop codon region of mRNAs [38]. The data included in this review suggested that VIRMA is mainly upregulated in various cancer types, and is associated with poor survival [20, 29–32, 42–44]. VIRMA has been shown to be upregulated and r associated with a more favorable survival in KIRC [41]. Further studies on the biological functions and mechanisms of VIRMA are required to identify its role in KIRC. Hou et al. showed that VIRMA was downregulated and predicted better prognosis in papillary thyroid carcinoma and acted as a tumor suppressor gene [52]. But the result was only from TCGA database and lacked the clinical confirm. VIRMA was also downregulated in KIRP and predicted better prognosis, which also need clinical confirm. By the m6A-dependent manner or the m6A-independent manner, VIRMA has been shown to promote cancer cell proliferation, apoptosis resistance, invasion, migration, and tumor progression through different pathways, including the TGF-β signaling pathway by targeting ID2, cell cycle pathway by targeting CDK1, CCAT1/2 pathway by upregulating its lncRNA stability, GATA3 pathway by upregulating pre-GATA3 mRNA degradation, and TNF pathway by targeting c-Jun [20, 29–32, 42–44]. Since circ_KIAA1429 can also promote tumor progression in hepatocellular carcinoma. VIRMA and circ_KIAA1429 both have great potential for clinical application by serving as a new treatment target. Therefore, more studies on VIRMA and m6A modification are needed to clarify the functions of VIRMA in the biological progress of various types of human cancer.

## Data Availability

The datasets analysed during the current study are available in the sanger box (http://sangerbox.com/) and GTEx database (http://commonfund.nih.gov/GTEx/). The mutation data during this study are included in Li’s published article (https://www.ncbi.nlm.nih.gov/pmc/articles/PMC6744659/).

## Abbreviations

m6A: *N*6-Methyladenosine
METTL3: Methyltransferase like 3
METTL14: Methyltransferase like 14
WTAP: Wilms tumor 1-associated protein
VIRMA: Vir like m6A methyltransferase associated
METTL16: Methyltransferase like 16
ZC3H13: Zinc finger CCCH-type containing 13
RBM15: RNA binding motif protein 15
ALKBH5: AlkB homolog 5
FTO: The fat mass and obesity-associated protein
IGF2BP: IGF2 mRNA binding proteins
eIF3: Eukaryotic translation initiation factor 3
HNRNP: Heterogeneous nuclear ribonucleoprotein
YTH: YT521-B homology domain-containing protein
3′-UTR: 3′-Untranslated region
CNV: Copy number variation
KIRC: Kidney renal clear cell carcinoma
KIRP: Kidney renal papillary cell carcinoma
KICH: Kidney Chromophobe
LGG: Brain lower grade glioma
GBM: Glioblastoma multiforme
BRCA: Breast invasive carcinoma
LUSC: Lung squamous cell carcinoma
LUAD: Lung adenocarcinoma
READ: Rectum adenocarcinoma
COAD: Colon adenocarcinoma
UCS: Uterine carcinosarcoma
UCEC: Uterine corpus endometrial carcinoma
OV: Ovarian serous cystadenocarcinoma
HNSC: Head and neck squamous carcinoma
THCA: Thyroid carcinoma
PRAD: Prostate adenocarcinoma
STAD: Stomach adenocarcinoma
SKCM: Skin cutaneous melanoma
BLCA: Bladder urothelial carcinoma
LIHC: Liver hepatocellular carcinoma
CESC: Cervical squamous cell carcinoma and endocervical adenocarcinoma
ACC: Adrenocortical carcinoma
PCPG: Pheochromocytoma and paraganglioma
SARC: Sarcoma
LAML: Acute myeloid leukemia
PAAD: Pancreatic adenocarcinoma
ESCA: Esophageal carcinoma
TGCT: Testicular germ cell tumors
THYM: Thymoma
MESO: Mesothelioma
UVM: Uveal melanoma
DLBC: Lymphoid neoplasm diffuse large B-cell lymphoma
CHOL: Cholangiocarcinoma
OS: Overall survival
DSS: Disease-specific survival
DFI: Disease-free interval
